# Fractionation of *Xanthium strumarium* L. foliage phenolics, *in-vitro* antioxidant activities, and *in-vivo* anti-diabetic potential

**DOI:** 10.3389/fchem.2023.1279729

**Published:** 2023-11-20

**Authors:** Asma Shaheen, Sumia Akram, Saima Sharif, Ayoub Rashid, Ahmad Adnan, Muhammad Mushtaq

**Affiliations:** ^1^ Department of Chemistry, Government College University Lahore-Pakistan, Lahore, Pakistan; ^2^ Division of Science and Technology, University of Education Lahore-Pakistan, Lahore, Pakistan; ^3^ Department of Zoology, Lahore College for Women University Lahore-Pakistan, Lahore, Pakistan

**Keywords:** *Xanthium strumarium*, L. Foliage, fractionations, phenolics, antioxidants, antidiabetics

## Abstract

**Introduction:** The present research aimed to fractionate *Xanthium strumarium* L. (XSL) foliage phenolics into a set of solvents and evaluate their antioxidant potential and *in-vivo* anti-diabetic activity against Alloxan monohydrate-induced diabetic mice.

**Methodology:** For this purpose, XSL foliage was fractionated into petroleum ether, ethyl acetate, ethanol, and water *via* orbital type shaking and tested for the presence of phenolics, and their antioxidant and antidiabetic potential.

**Results and discussion:** The results revealed that the ethyl acetate fraction of XSL foliage contained the highest amount of total phenolics 95.25 mg GAE/g of extract, followed by ethanol (65.14 mg GAE/g), petroleum ether (25.12 mg GAE/g), water (12.20 mg GAE/g), and XSL powder (69.13 mg GAE/g). At the end of treatment time (day 18 of oral administration of 400 mg/kg body weight of mice), the ethyl acetate fraction significantly (*p* ≤ 0.05) lowered blood glucose level (353 ± 10.6 to 220 ± 25.5 mg/dL) which might due to the elevated level of phenolic compounds in this fraction.

**Conclusion:** Overall, it can be speculated that ethyl acetate and ethanol may work efficiently for the enrichment of XSL phenolic without compromising their antidiabetic potential.

## Graphical Abstract

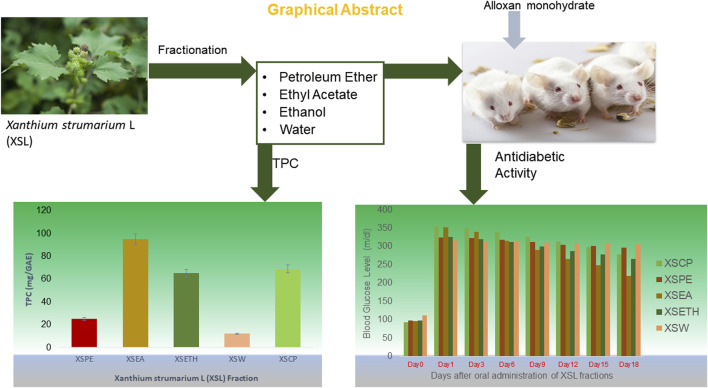

## 1 Introduction

Despite the prevalence and widespread use of anti-diabetic medications like metformin, sulfonylureas, thiazolidinediones, and insulin, diabetes ranks as a major global health concern. International Diabetic Federation estimates indicate that the prevalence of diabetes has increased to 463 million in 2019 and figures may rise to 578 (10.2%) and 700 million (10.9%) by 2030 and 2045, respectively (Akhtar et al., 2022). Additionally, it has been predicted that by 2030, diabetes will account for 3.3% of all fatalities worldwide, making it the seventh greatest cause of death (Kokil et al., 2015). For centuries herbs have been used to meet healthcare needs through medical procedures and traditional remedies. Traditional medicine employs at least 1200 different plant species for their potential biological activities and almost half of these have been studied for their phytochemistry, and antioxidant activities (Krupa et al., 2019; Mawoza et al., 2019).

*Xanthium strumarium* Linn (hereafter XSL) belongs to the Asteraceae family and is commonly identified as cocklebur, sheep bur, hedgehog bur weed, ditch bur, sea burdock, clot bur, and button bur by different civilizations in the world (Ghahari et al., 2017). The plant has habitats worldwide, but temperate zones are the areas of high prevalence, especially India, Russia, Iran, Australia, North Korea, Japan, Pakistan, America, South Africa, China, Eastern Asia, and some parts of South Asia. It frequently grows along roadsides, and on plains, hills, and mountains. The flowering period starts during July/August whereas fruits are usually ripe in September/October (Fan et al., 2019). The fruit as a whole clings to animal fur for its dispersal and is considered poisonous, however, it possesses cytotoxic, sedative diuretic, antitussive antimalarial, antispasmodic, anti-rheumatic antibacterial, and antifungal properties (Clayton et al., 2020). Few researchers have evaluated the antidiabetic potential of *X. strumarium* L. (XSL), but the majority of these studies utilized aqueous or alcoholic extracts of XSL foliage (Ahmad et al., 2016). An acute review of previously published research regarding phenolics reveals that the solubility of these compounds varies widely and similar can be speculated about the stability and biological functions of these bioactives. Keeping in view these facts, we have planned to fractionate XSL foliage phenolics into a range of solvents including petroleum ether, ethyl acetate, ethanol, and water and each fraction of XSL foliage was subsequently assessed for its total phenolic content (TPC), radical scavenging capacity, *in-vitro* antioxidant activity, and antidiabetic potential (*in-vivo*).

## 2 Materials and methods

The experimental work regarding the fractionation of XSL foliage was done in the Biochemistry laboratory of Government College University Lahore. The *in-vivo* trials associated with the present research were carried out at the animal house in the Lahore College for Women University’s Zoology Department. Formal approval was obtained from the institutional ethical committee (No. GCU-IIB-2596) dated 31 October 2022. Animals (Swiss albino mice) were supplied by F. Z traders, Lahore, Pakistan, and at the time of the experiment the average age and weight ranged from 4 to 8 weeks and 22–24 g, respectively. The chemicals and solvents used during the current study were of analytical grade and acquired from the companies Sigma Aldrich Chemical Co. (United States) and Merck (Germany).

### 2.1 Sample collection

The XSL foliage was collected during the month of June 2022 from a remote location in Pakistan’s District Narowal and was identified as rough cocklebur (voucher no. LCW -1016) by Dr. Shubnam Shaheen Associate Professor, Department of Botany, Lahore College for Women University Lahore.

### 2.2 Preparation of XSL fractions

The XSL foliage was air dried at 40°C after being cleaned with distilled water to remove dirt and contaminants. The dried XSL foliage was powdered prior to the fractionation with particle mesh sizes ranging from 24 to 60 sorted by electromagnetic vibrator for 15 min, packed in an opaque plastic bag, and stored. Petroleum ether, ethyl acetate, ethanol, and water were sequentially used as solvents during the fractionation process. A carefully-weighed 25 g of dried XSL foliage powder was placed in a 500 mL Erlenmeyer flask and shaken with 250 mL of petroleum ether for 12 h in an orbital shaker (Gallenkamp, UK) at 250 rpm under ambient conditions 25°C–30°C. The resultant aliquot was filtered using Whatman filter paper 1, and residues were mixed with the second extraction solvent, *i.e.*, ethyl acetate, and shaken under the above-mentioned conditions. The residues of step 2 and step 3 were subsequently subjected to ethanol- and water-based fractionation following the above-mentioned procedure. All four fractions were separately dried using a rotating evaporator (SB-651; EYELA, Tokyo, Japan) operated at 35°C and reduced pressure, weighed to calculate the percent yield of each fraction (g/100 g of XSL powder), and stored at −10°C until the required dosages were made. Finally, the amount of extractable bioactives (total extract) was calculated by adding the percent yield of each fraction.

### 2.3 Total phenolic content

The TPC in every fraction of XSL foliage powder was measured using Folin-Ciocalteu reagent as documented by (Elagdi et al., 2023). Gallic acid was processed as a positive control to express the results as milligrams of gallic acid equivalent (mg GAE)/g) of extract. The five different concentrations of the gallic acid standard (50, 40, 30, 20, and 10 mg/mL) and 1.0 mg/mL of each XSL foliage powder fraction were introduced into the test tube containing 2.5 mL of 10% (v/v). To the Folin-Ciocalteu reagent was added 2 mL of 7.5% (w/v) Na_2_CO_3,_ and the mixture was allowed to stand in the dark at room temperature for half an hour. The absorbance was measured at 760 nm using a Shimadzu 160-UV spectrophotometer. For each extract, all the calculations were done in triplicates.

### 2.4 DPPH assay

The radical scavenging potential of each XSL foliage fraction was estimated by using the method described by Villano et al. with minor changes (Villaño et al., 2007). In this assay, 0.1 mM solution of 1,1-diphenyl-2-picrylhydrazyl and 1.0 mg/mL of each XSL extract was prepared in HPLC grade methanol. The equal volumes 2.5 mL of DPPH solution and extract were incubated at room temperature for half an hour under dark, and at 517 nm absorbance was measured and compared with ascorbic acid acting as a standard. The free radical scavenging potential of the XSL fraction was expressed as %inhibition calculated by the following formula where As and Ac stand for the absorbance of the sample and control respectively. The IC_50_ (Conc. providing 50% inhibition) value is used to express the DPPH assay results. IC_50_ value can be measured by plotting a graph between the scavenging effect and the corresponding extract concentration. AAI (Antioxidant activity index) is used to measure the antioxidant potential and can be calculated by dividing the final DPPH concentration (µg/mL) by IC_50_ (µg/mL) (Scherer and Godoy, 2014).

$$\%\text{Inhibition}=[\left(\text{Ac}‐\text{As}\right)/\text{Ac}]\;\times 100$$

### 2.5 FRAP assay

The ferric-reducing ability of plasma (FRAP) comprising XSL foliage fractions was determined by using the strain and Benzie method with minor changes (Benzie and Strain, 1996). In this method, 300 µL distilled water was mixed in 100 µL of plant extract followed by the addition of 3 mL of frap reagent. The frap reagent was prepared by mixing 25 mL acetate buffer (0.3 M) at PH = 3.6 and 2.5 mL (10 mM) of TPTZ (2, 4, 6- tripyridyl-s-triazine) in hydrochloric acid (40 mM) and 2.5 mL of FeCl_3_ (20 mM). The mixture was incubated under ambient conditions (30°C) and absorbance was measured at 593 nm by a Shimadzu 160-UV spectrophotometer. Frap assay evaluates the antioxidant activity of plant extract by reducing metal ions using electron donation. Ascorbic acid was used as the standard and the FRAP value was measured by the following equation, Where A_O_ and A_S_ are the absorbance of the standard and sample respectively.

$$\text{Frap value }\left(\left(µm\text{ascorbate}/g\right)=\text{As}‐\text{Ao}/\text{Ao}\right.$$

### 2.6 Induction of diabetes in mice

For the animal study, 45 male albino mice were selected and were kept for 1 week of acclimatization. With a maximum of six animals per cage, the animals were housed in sterile polypropylene cages and kept at room temperature. The bedding material was sterile rice husk. These animals were housed in a controlled setting with respect to temperature (25°C), humidity (45%–57% range), and light period (12: 12-h dark-light cycle). Alloxan monohydrate was intraperitoneally injected at a dosage of 120 mg/kg to cause hyperglycemia in male albino mice that were more than 8 weeks old and weighed 22–24 g. The blood glucose levels were assessed by tail clip sampling 48 h following alloxan administration. When the blood sugar level exceeded 185 mg/dL, mice were considered diabetic (Nagappa et al., 2003). The animals were divided into seven groups each having six mice and the body weight and blood glucose (using glucometer) level of control and treated groups were monitored on days 1, 3, 6, 9, 12, 15, and 18 of treatment.

C: Mice who had not been given any drug or alloxan monohydrate (Normal control).

DC: Mice were given alloxan monohydrate (Diabetic control).

XSCP: Diabetic mice were administered with crude powder of XSL.

XSPE: Diabetic mice were administered a petroleum ether fraction of XSL.

XSEA: Diabetic mice were administered with ethyl acetate fraction of XSL.

XSETH: Diabetic mice were administered an ethanolic fraction of XSL.

XSW: Diabetic mice were administered with an aqueous fraction of XSL.

### 2.7 Acute toxicity studies

The process was completed in accordance with OECD (Organization for Economic Co-operation and Development) guidelines to check the toxicity (Ahmad et al., 2016). To investigate the acute toxicity, different doses of XSL foliage fractions were administered to male Swiss albino mice. The control group received the vehicle (normal saline only), whereas the treatment groups orally received plant fractions in dosages of 2 and 5 g/kg. The adverse effects were thoroughly monitored for 2 days after the dose was introduced. The body weight of the mice before and after administration, any sign of toxicity such as changes in the fur, skin, and eyes, as well as changes in the respiratory, circulatory, and central nervous systems, behavior patterns, signs of tumors, salivation, diarrhea, sleep, and coma were also noted.

### 2.8 Blood collection and determination of blood glucose

To determine the blood glucose level blood samples were collected from fasted mice on days 0, 1, 3, 6, 9, 12, 15, and 18 of the treatment. Blood was collected^1^ from the mouse tails by snipping with a sharp razor, and glucose level was measured by glucometer (Accu-Chek Active, Roche Limited, Pakistan)^2^ as described by Arya et al. (2012).

### 2.9 Statistical analysis

All the parameters including extract yield, TPC, and DPPH radical scavenging activity were measured in triplicate and the estimates were compared^3^ for significant difference *p* ≤ 0.05 by using Duncan’s multiple range^4^ test (MRT) (Tallarida and Murray, 1987).

## 3 Results and discussion

Table 1 shows the percentage yield^5^ of fractions of XSL foliage depending on the solvent polarity that significantly affected the fraction yield in the order of petroleum ether < ethyl acetate < ethanol < water. Fraction yield depends upon the solvent polarity and the difference in fraction yield in different solvents was due to the solubility of phytochemicals in the respective solvent (Dirar et al., 2019). TPC of different fractions of XSL in ethyl acetate was 95.25 ± 7.41 mg GAE/g, followed by ethanol 65.14 ± 7.06 mg GAE/g, petroleum ether 25.12 ± 7.06 mg GAE/g, and water 12.2 ± 5.80 mg GAE/g. The TPC in crude powder of XSL foliage was found to be 69.13 ± 6.01 mg GAE/g. In the previous studies TPC value of ethyl acetate and aqueous leave extract of XSL extracted by maceration and hot extraction method was 59.98 and 35.92 mg of GAE/g DW, respectively (Pillai and Thebe, 2023). The TPC value of ethanolic extract prepared from the aerial parts of XSL using the cold percolation method was determined 84.86 ± 5.13 mg of GAE/g DW (Ly et al., 2021). Similarly, aqueous and ethyl acetate fractions were obtained from 80% methanolic extract of XSL dried leaves using the cold percolation method, and their TPC values were determined as 75.24 ± 13.31 and 166.26 ± 27.98 mg of GAE/g DW (Guemmaz et al., 2018; Pillai and Thebe, 2023). In other studies ethyl acetate and 80% ethanol extract were extracted from XSL dried leaves by dynamic maceration, static maceration, and soxhlet method, and their TPC values were determined as 70.07 ± 1.6, 64.51 ± 1.0, 69.38 ± 1.3 mg GAE/g DW (Ethanol), 27.19 ± 1.0, 21.98 ± 3.6 and 23.19 ± 0.3 mg GAE/g DW (Ethyl acetate) respectively (Scherer and Godoy, 2014). No literature reported on petroleum ether fraction. The discrepancy in TPC value is due to factors like sample variety, sample quantity, type of extraction techniques, seasonal variation, extracted bioactive components, and geographic location.

**TABLE 1:** The XSL foliage fraction yield in various solvents and their Total Phenolic Content (TPC).

Fractions	% yield (w/w)	TPC (mg GAE/g)
XSPE^1^	0.033 ± 0.01^a^	25.12 ± 7.06^ab^
XSEA^2^	0.095 ± 0.02^a^	95.25 ± 7.41^c^
XSETH^3^	0.301 ± 0.07^b^	65.14 ± 7.06^b^
XSW^4^	0.402 ± 0.28^bc^	12.2 ± 5.80^a^
Total	0.828 ± 0.38^d^	197.71 ± 27.33^d^
Crude powder (XSCP^5^)	----	69.13 ± 6.01^bc^

The values are mean ± SD, of triplicate experiments, the different superscript letters over each range denote the significantly different (*p* ≤ 0.05) values.

### 3.1 Antioxidant activity

The DPPH assay evaluates the antioxidant’s scavenging capacity towards the DPPH radical. DPPH is a stable free radical having a blue color in alcoholic solution, while in the presence of antioxidants its reduction changes color to yellow. DPPH assay depends upon the antioxidant’s potential to donate hydrogen or electrons and is spectrophotometrically analyzed. Absorbance is inversely proportional to the antioxidant potential which is directly proportional to % inhibition. Table 2 shows the antioxidant activity of petroleum ether (XSPE), ethyl acetate (XSEA), ethanol (XSETH), and water (XSW) fractions of XSL. The IC_50_ value decreased in the order of 101.20 > 88.02 > 54.60 > 46.11 and the antioxidant index value (AAI) increased in the order of 1.87 > 0.90 > 0.28 > 0.05 respectively. In the present study the ethyl acetate fraction has the lowest IC_50_ value followed by the ethanol fraction, while in the previous literature the IC_50_ value of different solvent extracts of *Xanthium strumarium* aerial parts has been determined. IC_50_ values of aqueous and ethyl acetate extracts (obtained by maceration and hot percolation method) have been determined at 2465.21 and 1856.02 μg/mL, respectively (Kim et al., 2005; Guemmaz et al., 2018; Pillai and Thebe, 2023). Similarly Aqueous and ethyl acetate fractions of 80% methanol extract of dried XSL leaves obtained by cold percolation method have demonstrated IC_50_ values 46.00 ± 0.0006, 17.00 ± 0.0004 μg/mL respectively (Pillai and Thebe, 2023). In another study, 80% ethanol and ethyl acetate extract prepared from XSL dried leaves by dynamic maceration, static maceration, and soxhlet method and their IC_50_ values were determined as 53.01 ± 1.20, 47.83 ± 1.40, 53.34 ± 1.52 μg/mL (Ethanol), 369.83 ± 13.58, 346.35 ± 16.50, and 423.97 ± 22.27 μg/mL (Ethyl acetate) in the DPPH assay (Scherer and Godoy, 2014). There was no literature reported on petroleum ether fraction. The variation in the IC_50_ in the present study and previous reports might be due to the above-mentioned factors as discussed before.

**TABLE 2:** DPPH Radical scavenging activity of different fractions of XSL foliage powder.

Fractions	IC_50_ μg/mL	AAI
XSPE	88.02^c^	0.28^b^
XSEA	46.11^a^	1.87^d^
XSETH	54.60^b^	0.90^c^
XSW	101.20^d^	0.05^a^

The values are mean ± SD, of triplicate experiments, the different superscript letters over each range denote the significantly different (*p* ≤ 0.05) values.

FRAP assay evaluates the antioxidant potential of antioxidants in terms of the reduction of ferric to ferrous ions. Ascorbic acid is used as standard and the values of reducing potential of antioxidants are compared to it. With the increase of antioxidant concentration, absorbance increases, and the resulting reducing power also increases. The frap value will be greater the higher the antioxidant potential. Table 3 depicts the Frap values and absorbance of different fractions of XSL foliage. Ethyl acetate has greater absorbance with FRAP value of 0.238 ± 0.06 (µm ascorbate/g) followed by ethanol (0.212 ± 0.07), petroleum ether (0.194 ± 0.04) and water (0.179 ± 0.01) µm ascorbate/g. In the literature cited the ferric reducing capacity of ethyl acetate and water extracts of XSL have been determined to be 0.996 ± 0.101 and 0.412 ± 0.009 µm ascorbate/g at a concentration of 100 μg/mL (Kim et al., 2005; Pillai and Thebe, 2023). While the FRAP value in this research work was measured at 30 μg/mL. The FRAP values of ethanol and petroleum ether fractions were not reported in the literature.

**TABLE 3:** FRAP Assay of different fractions of XSL foliage powder.

Sample	Absorbance	Frap value (µm ascorbate/g)
XSPE	0.75	0.194 ± 0.04
XSEA	1.19	0.238 ± 0.06
XSETH	0.98	0.212 ± 0.07
XSW	0.59	0.179 ± 0.01

### 3.2 Body weight

Table 4 elaborates on the difference in body weight between the experimental and control group animals after treatment with different fractions of XSL foliage. The mice administered alloxan monohydrate (120 mg/kg) lost body weight from 1% to 3% which was averted by the treatment of different fractions and powdered samples of the selected plant. The control group animals (C) which use only the vehicle shows an increase in body weight from 21.5 ± 2.12 to 24.75 ± 1.77 (14%) while diabetic control (DC) loss in body weight from 23.50 ± 0.71 to 21.05 ± 0.78 (10%) on day 18 days.

**TABLE 4:** Effect of different fractions of XSL on body weight in alloxan-induced diabetic mice.

Groups	Doses (mg/kg)	Days after treatment							
		0	1	3	6	9	12	15	18
C		21.50 ± 2.12	21.65 ± 2.05	21.90 ± 1.98	22.20 ± 1.84	22.55 ± 1.91	22.90 ± 1.84	23.75 ± 1.77	24.75 ± 1.77
DC		23.50 ± 0.71	23.30 ± 0.57	23.25 ± 0.78	22.80 ± 0.85	22.45 ± 0.78	22.00 ± 0.71	21.45 ± 0.78	21.05 ± 0.78
XSCP	400	23.00 ± 2.83	22.40 ± 2.69	22.30 ± 2.69	22.75 ± 2.76	23.05 ± 2.62	23.25 ± 2.62	23.60 ± 2.69	24.00 ± 2.82
XSPE	400	23.50 ± 0.71	23.15 ± 0.50	23.00 ± 0.57	22.50 ± 0.71	22.65 ± 0.64	22.80 ± 0.57	22.90 ± 0.57	23.00 ± 0.57
XSEA	400	21.50 ± 0.71	21.15 ± 0.64	21.60 ± 0.71	21.95 ± 0.64	22.50 ± 0.71	22.85 ± 0.64	23.30 ± 0.71	24.00 ± 0.71
XSETH	400	23.50 ± 0.71	23.05 ± 0.64	22.95 ± 0.64	23.15 ± 0.78	23.55 ± 0.78	23.65 ± 0.78	23.75 ± 0.78	24.15 ± 0.64
XSW	400	22.75 ± 1.06	22.55 ± 1.06	22.30 ± 0.99	22.3 ± 0.71	22.40 ± 0.71	22.45 ± 0.78	22.50 ± 0.71	22.70 ± 0.71

The values are mean ± SD, of triplicate experiments.

The animals in the XSCP sample lost body weight by 23 ± 2.83 to 22.40 ± 2.69 (3%) on the first day and after receiving with powdered sample increased by 24 ± 2.82 (7%) on the last day of the trial. The XSPE and XSEA animals reduced their body weight from day 1 23.50 ± 0.71 to 23.15 ± 0.50 (1%) and 21.50 ± 0.71 to 21.15 ± 0.64 (2%) respectively and after being treated with petroleum ether and ethyl acetate fractions, the weight of XSPE and XSEA animals increased up to 23 ± 0.57, 24 ± 0.71 (1% and 13%) of their initial body weight. The animals treated with the ethanol and water fractions showed body weight reduction of 23.5 ± 0.71 to 23.05 ± 0.64 (2%), 22.75 ± 1.06 to 22.55 ± 1.06 (1%), and after treatment increased up to 24.15 ± 0.64, 22.70 ± 0.71 (5% and 1%) respectively. The results shown are based on the mean of six mice and the percentage change indicates the change from day 1 to the end of the study (day 18).

### 3.3 Antidiabetic activity

The blood glucose level of diabetic mice (CD) was significantly (*p* ≤ 0.05) higher as compared to normal mice (C) and the effect of powdered sample and different fractions of XSL on diabetic mice was studied. Data assembled in Table 5 shows the blood glucose levels of the control and treatment groups on days 1, 3, 6, 9, 12, 15, and 18 of treatment. The blood glucose level of C (control group), who simply used a vehicle, did not vary significantly (*p* ≤ 0.05) during the treatment time (as shown by subscript and superscript letters). However, the blood glucose level of diabetic mice (DC) was significantly (*p* ≤ 0.05) higher as compared to the control group. Fasting mean blood glucose level of diabetic control DC and XSCP on day one after being diabetic was 345 ± 7.07 mg/dL, and 355 ± 7.07 mg/dL, respectively. On day 18 the blood glucose level of DC (diabetic control) increased up to 364 ± 2.12 mg/dL, while oral administration of powdered sample to XSCP significantly (*p* ≤ 0.05) decreased the blood glucose level to 278 ± 10.6 mg/dL (22%). On day one, blood glucose levels of diabetic-induced mice of XSPE, XSEA, XSETH, and XSW were measured as 325 ± 21.2 mg/dL, 353 ± 10.6 mg/dL, 327 ± 9.2 mg/dL, and 316 ± 8.5 mg/dL, respectively. There was no substantial decrease in the blood glucose level of the XSPE group (treated with petroleum ether) observed and two mice out of six died on days 8 and 13 of the treatment, while on day 18 the mean blood glucose level of the remaining mice decreased upto 297 ± 26.2 mg/dL (9%). Animals treated with ethyl acetate (XSEA) showed a substantial decrease in the blood glucose level from day 1 353 ± 10.6 mg/dL to 220 ± 25.5 mg/dL (38%) on day 18. The blood glucose levels of animals treated with ethanol (XSETH) and water (XSW) fractions on the day 18 were 266 ± 15.6 mg/dL and 306 ± 14.1 mg/dL respectively.

**TABLE 5:** Blood Glucose Level of alloxan-induced diabetic mice at different intervals after XSL fractions administration.

Groups	Dose (mg/kg)	Days after treatment							
		0	1	3	6	9	12	15	18
C		103 ± 9.90^a^_a_	95.5 ± 0.71^a^_a_	97 ± 4.24^a^_a_	91 ± 1.41^a^_a_	94 ± 2.83^a^_a_	96.5 ± 2.12^a^_a_	93 ± 4.24^a^_a_	97 ± 1.41^a^_a_
CD		105 ± 7.07^a^_a_	345 ± 7.07^d^_d_	348 ± 5.66^d^_d_	350 ± 5.66^d^_d_	355 ± 7.07^d^_d_	358 ± 5.66^d^_d_	363 ± 3.54^d^_d_	364 ± 2.12^d^_d_
XSCP	400	93.5 ± 0.71^a^_a_	355 ± 7.07 ^d^_d_	350 ± 7.07 ^d^_d_	339 ± 8.49 ^c^_c_	327 ± 9.20 ^c^_c_	313 ± 11.3^c^_c_	299 ± 4.95 ^b^_b_	278 ± 10.6 ^b^_b_
XSPE	400	97.5 ± 3.54^a^_a_	325 ± 21.2 ^d^_c_	323 ± 21.9 ^cd^_b_	318 ± 21.9 ^cd^_b_	313 ± 21.9 ^cd^_b_	305 ± 21.2 ^c^_b_	301 ± 22.6^c^_b_	297 ± 26.2 ^c^_b_
XSEA	400	96 ± 2.73^a^_a_	353 ± 10.6 ^d^_d_	341 ± 10.6 ^d^_cd_	316 ± 7.78 ^d^_b_	291 ± 5.66 ^c^_b_	267 ± 23.3^b^_b_	250 ± 23.3^b^_b_	220 ± 25.5 ^b^_b_
XSETH	400	99 ± 4.95^a^_a_	327 ± 9.2 ^d^_c_	320 ± 7.1 ^d^_bc_	312 ± 4.95 ^d^_c_	300 ± 7.1^d^_b_	288 ± 10.6^c^_b_	278 ± 13.4 ^c^_b_	266 ± 15.6^c^_b_
XSW	400	112 ± 4.24^a^_a_	316 ± 8.5 ^d^_d_	314 ± 9.90 ^d^_d_	315 ± 12.73 ^d^_d_	310 ± 14.1 ^c^_b_	309 ± 14.4 ^c^_b_	308 ± 14.1^c^_c_	306 ± 14.1 ^c^_c_

The values are mean ± SD, of triplicate experiments, the different letters over each range denote variation (*p* ≤ 0.05) in blood glucose level at various intervals (superscripted letters) and type of fraction administered (subscript letters).

Ethanol fraction lowers the blood glucose level by up to 19% while water fraction decreases it by only 3%. Overall, it can be claimed that the ethyl acetate fraction of XSL (XSEA) contains substantial amounts of phenolic bioactive antioxidants which decreased the blood glucose level of diabetic mice. However, more comprehensive studies with a larger number of mice might be helpful to generalize these findings.

In the previous studies, the aqueous leaf extract of XSL was also used as an antidiabetic agent at a dose of 500 mg/kg and 250 mg/kg body weight respectively in alloxan-induced diabetic mice for 10 days and a significant reduction in blood glucose level was observed at the high dose of XSL aqueous extract (Mouhamad, 2022). Similarly, methanolic extract of XSL leaves prepared by the soxhlet apparatus was used to determine the antioxidant potential (*in-vitro*) and antidiabetic potential (*in-vivo*) in albino rats by oral administration at a dose of 200 mg/kg and 400 mg/kg body weight for 9 days (Umer et al., 2016). In another study hypoglycemic effect of ethanolic extract of XSL and its isolated compound were evaluated in alloxan-induced diabetic mice for 14 days and compared with Glibenclamide 10 mg/kg b. w; an antidiabetic drug (Ahmad et al., 2016). Leaf and fruit methanolic extract of *X. strumarium* obtained by the soxhlet method was used to measure the antidiabetic effect in diabetic mice by oral administration of 400 mg/kg b. w of extract for a period of 24 days (Harikumar et al., 2012). Methanolic extract of XSL stem was used to evaluate the hypoglycemic effect at doses of 100 mg/kg and 200 mg/kg body weight and antidiabetic potential was compared with Glibenclamide 0.6 mg/kg in Streptazocin-induced diabetic rats (Narendiran et al., 2011). By surveying the literature, it can be observed that there is no research work published about crude foliage fractionation and comparison of their antioxidant, antidiabetic potential, and variation in body weight. In the present research work, four fractions of XSL were obtained and their total phenolic content (TPC), antioxidant, and antidiabetic potential were studied. The antidiabetic potential of ethyl acetate fraction (XSEA) fractions was comparable with standard Glibenclamide. By comparing results observed by all fractions, it has been observed that the XSEA fraction showed high TPC value, antioxidant potential and antidiabetic effect followed by the ethanol, petroleum ether, and aqueous fractions.

## 4 Conclusion

The aim of the current research was to assess the antidiabetic potential of different fractions of XSL foliage on diabetic mice. After 18 days of constant administration of XSL fractions, the blood glucose level of mice significantly decreased. These verdicts reinforced the traditional usage of the XSL foliage as an antidiabetic mediator as well as for the treatment of a number of ailments (Rahman et al., 2022). In order to boost insulin release from pancreatic cells, the conventional medication Glibenclamide has been used to treat diabetes for several decades. Several plant extracts were reported to exhibit hypoglycemic effects through stimulatory actions on insulin release, which is a possible pathway by which plant extracts lower blood glucose levels. This mechanism involves elevating the pancreatic insulin secretion from islets of Langerhans β cells or through its breakdown from the confined form (IfedibaluChukwu et al., 2020). According to some reports, other plants may also influence blood glucose levels by stimulating insulin release. Some wild herbs have chemical components that have hypoglycemic effects. There is evidence that certain types of plant-derived compounds, including carboxyatractyloside, chlorogenic acid, caffeic acid, and other phenolic compounds lower blood glucose levels. It has been found that plant’s extracts and metabolites have pharmacological effects. The results obtained supported the use of XSL in conventional medical system to cure diabetes. To determine the precise pathway of the anti-diabetic activity of the XSL fractions, a more comprehensive pharmacological and chemical research with large number of subjects is required.

## Data Availability

The original contributions presented in the study are included in the article/Supplementary material, further inquiries can be directed to the corresponding authors.

## Footnotes

1 Petroleum ether fraction of *X. strumarium* L. Foliage.

2 Ethyl acetate fraction of *X. strumarium* L. Foliage.

3 Ethanol fraction of *X. strumarium* L. Foliage.

4 Water fraction of *X. strumarium* L. Foliage.

5 Crude powder of *X. strumarium* L. Foliage.
